# Formation and Characterization of Supported Lipid Bilayers Composed of Hydrogenated and Deuterated *Escherichia coli* Lipids

**DOI:** 10.1371/journal.pone.0144671

**Published:** 2015-12-11

**Authors:** Tania Kjellerup Lind, Hanna Wacklin, Jürgen Schiller, Martine Moulin, Michael Haertlein, Thomas Günther Pomorski, Marité Cárdenas

**Affiliations:** 1 Nano-Science Center and Department of Chemistry, University of Copenhagen, Copenhagen, Denmark; 2 European Spallation Source ESS AB, Lund, Sweden; 3 Institute of Medical Physics and Biophysics, Faculty of Medicine, University of Leipzig, Leipzig, Germany; 4 Institut Laue-Langevin, Life Science Group, Grenoble, France; 5 Centre for Membrane Pumps in Cells and Disease—PUMPKIN, Department of Plant and Environmental Sciences, University of Copenhagen, Copenhagen, Denmark; 6 Malmoe University, Department of Biomedical Sciences, Health & Society, 20500 Malmoe, Sweden; UC Santa Barbara, UNITED STATES

## Abstract

Supported lipid bilayers are widely used for sensing and deciphering biomolecular interactions with model cell membranes. In this paper, we present a method to form supported lipid bilayers from total lipid extracts of *Escherichia coli* by vesicle fusion. We show the validity of this method for different types of extracts including those from deuterated biomass using a combination of complementary surface sensitive techniques; quartz crystal microbalance, neutron reflection and atomic force microscopy. We find that the head group composition of the deuterated and the hydrogenated lipid extracts is similar (approximately 75% phosphatidylethanolamine, 13% phosphatidylglycerol and 12% cardiolipin) and that both samples can be used to reconstitute high-coverage supported lipid bilayers with a total thickness of 41 ± 3 Å, common for fluid membranes. The formation of supported lipid bilayers composed of natural extracts of *Escherichia coli* allow for following biomolecular interactions, thus advancing the field towards bacterial-specific membrane biomimics.

## Introduction

Model lipid membranes with controlled and tunable physicochemical properties are widely used as biosensors for studying interactions with small biomolecules.[1–4] At the moment there are few good models for natural cell membranes, and cell properties are typically modeled using simple (phospho)lipid bilayers composed of just one or a few selected lipid classes. This is particularly true for studies involving techniques that require planar supported lipid bilayers and *in situ* measurements.[5–10] Indeed, many studies of antimicrobial interaction involve simple single-lipid membranes[5–7, 10] based on phosphocholines, a zwitterionic lipid class abundant in mammalian cells.[11] This is a consequence of the complexity of the natural bacterial membrane composition and the difficulties connected with forming and studying such complex lipid mixtures in a reproducible manner. Therefore, to date, there are very few publications where complex lipid extracts of e.g. *Escherichia coli*[12–15] have been deposited on a solid support. However, complex lipid mixtures of both hydrogenated and deuterated yeast extracts have recently been successfully deposited and characterized, showing a clear advancement in this field.[16]

**Fig 1:**
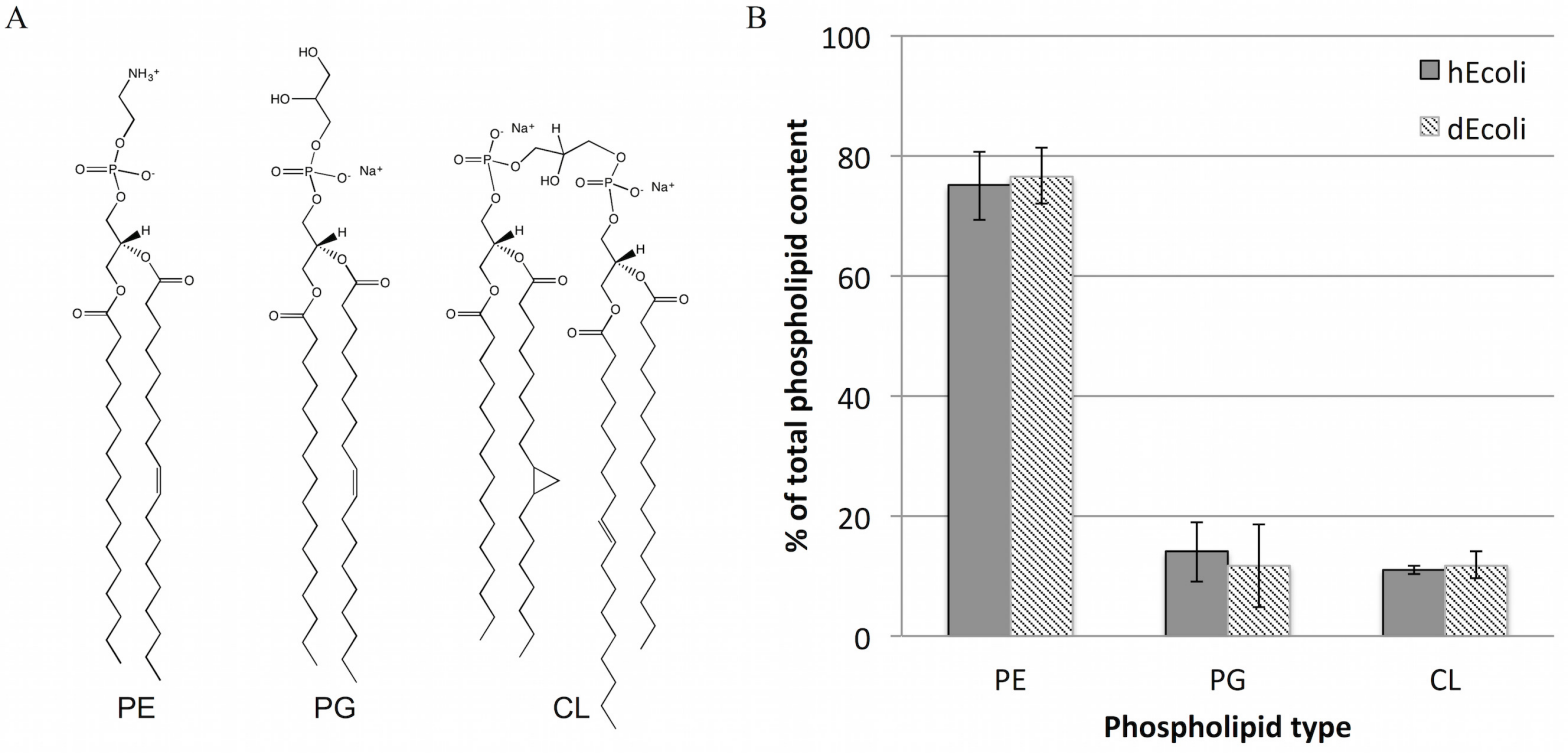
Lipid composition of total lipid extracts from hEcoli and dEcoli. A: Molecular structures of the most abundant species detected by MALDI-TOF mass spectrometry (see S1 Fig). B: Phospholipid composition of hEcoli (solid bars) and dEcoli (striped bars). Results are the means ± S.D. from two independent experiments. PE, phosphatidylethanolamine; PG, phosphatidylglycerol; CL, cardiolipin.

Ideally, reconstituted natural *E*. *coli* cell membranes should work as a bacterial model cell system containing the full range of biological lipids. The envelope of *E*. *coli* and other Gram-negative bacteria is composed of two cellular membranes that are separated by a thin layer of peptidoglycan. These are known as the inner (cytoplasmic) membrane (IM) and the outer membrane (OM).[17, 18] The phospholipid ratio in these two membranes is slightly different[19–21] but the most important difference between the membranes is the presence of lipopolysaccharide (LPS) in the outer leaflet of the OM.[18, 22] Extraction of bacterial lipids is typically done by the use of a mixture of chloroform and methanol.[23] The LPS and non-polar lipids are thus present in this total lipid extract.[24] By precipitation of the total extract with acetone and extraction with diethyl ether, the neutral and non-polar lipids including LPS can be removed resulting in a polar lipid extract. The *E*. *coli* lipid extract contains three main phospholipid classes, phosphatidylethanolamine (PE), phosphatidylglycerol (PG) and cardiolipin (CL) carrying predominantly C16:0, cyc17:0 and C18:1 acyl chains (for examples see Fig 1A).[24] The majority of the lipids in *E*. *coli* membranes belong to the PE class but the anionic PG and CL lipids are also present at relatively high fractions, effectively making the membranes net negatively charged.[24] Moreover, CL is an atypical lipid due to its four rather than two acyl chains and its comparatively small headgroup, which carries two negative charges. The presence of CL in lipid membranes can thus significantly alter their structural (lipid packing) and physical properties (charge density, phase transition temperature etc.). Indeed, this lipid has been shown to accumulate in the high curvature regions of *E*. *coli* membranes[25] and it plays a key role in antimicrobial peptide interaction.[26] To simplify bacterial membrane mimetic systems, CL is often omitted,[27–31] but in recent years the importance of this lipid in bacterial cell membranes and the significance of its presence in membrane mimics has become apparent[32–34]. Thus, as compared to simple lipid mixtures made from a few synthetic lipids, biomimetic bilayers composed of the *E*. *coli* total lipid extract represent an improved model of the *E*. *coli* membrane, in particular of the OM due to the presence of LPS.[35]

There are several methods to study lipid membranes and bio-molecular interactions, one of which is through the formation of supported lipid bilayers (SLBs) on surfaces. SLBs can be made in different ways using methods including lipid-detergent micelles,[36, 37] Langmuir-Blodgett deposition[38] and vesicle fusion[39–41]. The formation of SLBs by vesicle fusion is desirable due to the simplicity and reproducibility of the method and the advantage of depositing membranes *in situ* using a flow cell. However, the fusion of lipid vesicles and spreading of a bilayer on a solid support is not always straightforward and several experimental parameters must be considered and optimized for a specific lipid mixture in order to achieve complete bilayer formation. The properties of the solid support and the bulk solution are critical for successful bilayer formation especially for complex lipid/fatty acyl compositions and for lipid mixtures containing charges, such as bacterial lipids.[15] The surface charge, flow rate, deposition temperature, vesicle size, ionic strength, pH and the presence and concentration of divalent cations are all factors that determine these processes. Indeed, a fusion promoter in the form of divalent cations (typically salts of Ca^2+^ or Mg^2+^) is often needed for lipid vesicles with a net negative charge to adsorb to an anionic SiO_2_ or mica surface.[15, 42, 43] These ions act as bridges between the vesicles and the support, enabling the attachment of vesicles that, under optimal conditions, fuse and spread to form a planar membrane.

Here we present a study of the formation and structure of supported lipid bilayers composed of natural total lipid extracts from *E*. *coli*. We study the formation of small unilamellar vesicles by dynamic light scattering (DLS) and show that by optimizing the experimental conditions, SLBs can be formed *in situ* by vesicle fusion. We performed a systematic study and characterization of the quality and structure of such natural lipid bilayers using a range of complementary surface sensitive techniques including dissipation enhanced quartz crystal microbalance (QCM-D), neutron reflection (NR) and liquid atomic force microscopy (AFM). Furthermore, we characterized the lipid headgroup composition of hydrogenated and fully deuterated total *E*. *coli* lipid extracts and we show that supported lipid bilayers can be formed in both cases presenting similar structures. The deuterated bilayers are valuable tools for studying drug interactions by NR due to the increased contrast with biomolecules. Our results clearly show that bacterial lipid bilayers composed of the complete natural complex mixture of lipids found in *E*. *coli* can be deposited *in situ* by vesicle fusion. This is an important step towards constructing sensors coated with biomimetic membranes containing the full complexity of *E*. *coli* membranes that could lead to improved systematic studies of antimicrobial drug activity toward bacteria.

## Materials and Methods

All chemicals and solvents including D_2_O (99.9 atom% D) were obtained (in the highest commercially available purity) from Sigma-Aldrich (Brøndby, Denmark) and used as supplied unless stated otherwise. *E*. *coli* total lipid extract was purchased form Avanti Polar Lipids Inc. (Alabaster, AL, USA). Deuterated minimal medium was composed of 6.86 g/l (NH_4_)_2_SO_4_, 1.56 g/l KH_2_PO_4_, 6.48 g/l Na_2_HPO_4_·2H_2_O, 0.49 g/l diammoniumhydrogen-citrate, 0.25 g/l MgSO_4_·7H_2_O, 5g/l d_8_-glycerol (Euriso-Top, Saclay, France), 1.0 ml/l of a salt mix (0.5 g/l CaCl_2_·2H_2_O, 16.7 g/l FeCl_3_·6H_2_O, 0.18 g/l, ZnSO_4_·7H_2_O, 0.16 g/l CuSO_4_·5H_2_O, 0.15 g/l MnSO_4_·4H_2_O, 0.18 g/l CoCl_2_·6H_2_O, 20.1 g/l EDTA) in D_2_O. Luria Broth (LB) medium was composed of 10 g tryptone, 5 g yeast extract, 10 g NaCl per 1 L medium. Ultrapure water (with a resistivity of 18.2 MΩ cm) was used for all cleaning procedure and preparation of TRIS buffer (10 mM TRIS-HCl, 100 mM NaCl, pH 7.4). Hellmanex (Hellma Analytics GmbH & Co. KG) 2% was used for all cleaning procedures. Homemade solid-liquid flow cells were used for neutron reflection. The silicon blocks were 60×80×25 mm^3^ single crystals cut along the (111) plane.

### Production of h/d *E*. *coli* biomass

*E*. *coli* BL21 cells were grown in LB medium. After inoculation, the cultures were incubated overnight at 37°C with shaking. The preparation of a deuterium-adapted BL21 *E*. *coli* strain was performed by a multi-stage adaptation process.[44] After overnight incubation at 30°C, cells were streaked on solid agar plates containing 15 g/L agar and fully deuterated Enfors medium as described previously.[44] Following incubation for 30 h at 30°C, cells were transferred to a flask containing 10 mL deuterated minimal medium. This overnight culture was again incubated at 30°C. The adaptation to growth in D_2_O was established after four cycles of dilution and growth in 10 mL deuterated minimal medium. A pre-culture of 100 mL adapted cells were used to inoculate 1.5 L of deuterated minimal medium containing d_8_-glycerol (fully deuterated glycerol) as the carbon source in a 3 L fermenter (Labfors, Infors). Cells were grown to an optical density (OD_600_) of about 18 and harvested by centrifugation (10,000 g, 10 min, 4°C).

### Lipid extraction

Total lipids were extracted by a modified method of Bligh and Dyer[23]. In short, cell pellets were washed with H_2_O, re-suspended (5 ml H_2_O/g of cell paste) and sonicated (3 × 1 min pulses at about 20% power using a Branson Sonifier^®^ 450 Sonicator). Methanol (2.2 mL) and chloroform (1 mL) were added per 1-mL cell suspension aliquot. After 30 min at 25°C phase separation was induced by addition of chloroform (1 mL) and H_2_O (1 mL) followed by centrifugation (800 × g, 10 min, 4°C). The lower chloroform phase was collected, and the upper phase was twice re-extracted with 1 ml of chloroform. The chloroform phases were combined, dried using a nitrogen stream and stored at -20°C.

### Lipid analysis

Phospholipids were separated by two-dimensional thin layer chromatography (2D-TLC) using 10 × 10 cm glass plates coated with silica (silica gel 60, Merck). In the first chromatographic dimension, the TLC plates were developed in chloroform:methanol:water (65:25:4, v/v), dried thoroughly, and run in the second chromatographic dimension using chloroform:methanol:acetic acid (65:26:10, v/v). Lipids were visualized under ultraviolet (UV) light after staining with primuline (0.005% in acetone/water, 8/2; v/v), and identified by comparison with standards. For further analysis, lipid spots from primuline-stained TLC plates were scraped off and extracted three times with 100 μL chloroform/methanol/0.9% aqueous NaCl (1/1/1, v/v/v). All MALDI-TOF mass spectra were acquired on an Autoflex I mass spectrometer (Bruker Daltonics, Bremen, Germany) with ion reflector. Since all the phospholipids of interest are negatively charged negative ion mode spectra were exclusively recorded by using 9-aminoacridine (10 mg/ml in isopropanol/acetonitrile (60:40, v/v)). The samples were mixed 1:1 (v/v) with the corresponding matrix solutions. Further details are available in reference [45]. The total phospholipid content was determined by digesting lipids in deionized water and perchloric acid for 1 h at 180°C followed by addition of ammonium molybdate and ascorbic acid. After further heating to 80°C for 10 min, the samples were cooled and the absorbance was read at 812 nm to quantify the total amount of lipid phosphorus as previously described.[46]

### Liposome preparation

*E*. *coli* lipids dissolved in chloroform were dried under a stream of nitrogen and the resulting lipid film was vacuum desiccated overnight in order to remove any remaining organic solvent and stored at -18°C until use. Lipid films were re-suspended in TRIS buffer to a concentration of 200 μg/mL if not indicated otherwise and were left to hydrate for at least one hour at 50°C. Small unilamellar vesicles (SUVs) were prepared by sonication at 50°C. Bath sonication was performed for at 50°C during 1 h. Tip sonication was performed in a water bath at 50°C for 20 min with a 5 s on/off duty. In the latter case, SUVs presented an average hydrodynamic radius of 45 ± 5 nm, as determined by dynamic light scattering. Immediately prior to use the vesicles were diluted 1:1 with TRIS buffer containing 4 mM CaCl_2_ (for optimum conditions). For hot depositions the samples were kept at 50°C until use.

### Dynamic Light Scattering (DLS)

An ALV-5000 goniometer setup (ALV-GmbH, Langen, Germany) was used for DLS measurements at 90° angle. A 633 nm diode-pumped Nd:YAG solid-state Compass-DPSS laser light source (COHERENT, Inc., Santa Clara, CA) was used. The temperature was controlled at 25°C ± 0.1°C. The data was fitted by the regular fitting procedure using the ALV software and are presented as unweighted size distributions.

### Quartz crystal microbalance with dissipation monitoring

QCM-D was performed with the Q-SENSE E4 system (Q-Sense, Västre Frölunda, Sweden). The sensor crystals used were silicon oxide, 50 nm—purchased from Q-Sense. For cleaning, the sensor surfaces were placed in 2% Hellmanex for 10 min followed by thorough rinsing in absolute ethanol and ultrapure water. The surfaces were dried in a stream of nitrogen and oxidized in a UV-ozone chamber (BioForce Nanosciences, Inc., Ames, IA) for 10 min in order to remove molecular levels of contamination. O-rings were placed in 2% Hellmanex for 10 min followed by careful rinsing in ultrapure water and drying in a stream of nitrogen. The sample cells were quickly assembled to avoid contamination. Before performing any measurements the instrument temperature was set to 25°C and allowed to equilibrate. The fundamental frequency and six overtone frequencies (3rd, 5th, 7th, 9th, 11th, 13th) were found and a stable baseline was recorded. TRIS buffer supplemented with 2 mM CaCl_2_ was introduced in the flow cells using a peristaltic pump (Ismatec IPC-N 4) at a flow rate of 100 μL/min. The temperature was increased to 50°C and left to equilibrate before lipid introduction. Lipid vesicles, in a concentration of 100 μg/ml (50–250 μg/ml for the optimization measurements), were pumped into the cells at a flow rate of 100 μL/min.

### Neutron Reflection

The polyether ether ketone (PEEK) parts of the sample cells were cleaned by sonication in three 10 min cycles of Hellmanex 2% and ultrapure water. Silicon (111) surfaces were cleaned for 15 min in 5:4:1 H_2_O/H_2_SO_4_/H_2_O_2_ at 80°C and rinsed thoroughly with ultrapure water. The reflectometers FIGARO[47] and D17[48] at Institute Laue-Langevin (Grenoble, France) were used to record time of flight reflectivity using neutron wavelengths between 2–30 Å and two angles of incidence (FIGARO: 0.624° and 3.78°, D17: 0.8° and 3.2°). In all experiments the temperature was controlled using a water bath fixed at either 25 or 50°C. The vesicles were equilibrated in the cell for ~1 h before rinsing with TRIS supplemented with 2 mM CaCl_2_ following rinsing with calcium-free TRIS buffer. The bilayers were characterized at the temperature given in the text using at least two water contrasts (D_2_O, H2O and their mixtures). All NR profiles obtained in this study were analyzed using the Motofit package[49], which uses the Abeles optical matrix method to calculate the reflectivity of thin layers. The SiO_2_ surface was fitted to a two-layer model (Si–SiO_2_), and the bilayers were fitted to a one (dEcoli) or three (hEcoli) layer model, the latter consisting of head groups–lipid tails–head groups. The water distribution in the three-layer model was restricted to maintain identical mean molecular areas for the head groups and the lipid tails to keep a physically realistic bilayer model. In this case a standard lipid volume of POPC head and tail groups[50, 51] were used to calculate an estimated area per molecule, as this lipid is both very well-characterized[52] and has 16:0 and 18:1 acyl chains like the majority of the *E*. *coli* lipids. An additional water layer in between the lipid bilayer and the SiO_2_ was necessary in order to fit the data. The fitting errors of the headgroup and tail thicknesses and solvent fractions were determined by the quality of the fits.[53] The SLD for the lipids was fitted around the calculated SLD based on the composition determined in Fig 1. The headgroups exchange protons with the solvent giving rise to a slight change in headgroup SLD, and this was taken into account in the headgroup region of the three-layer fit assuming the composition obtained in Fig 1.

### Atomic Force Microscopy

AFM measurements were carried out on a Nanoscope IV multimode AFM (Veeco Instruments Inc.). Images were generated in the PeakForce QNM (quantitative nanomechanical property mapping) mode with a silicon oxide tip (Olympus microcantilever OTR8 PS-W) having a spring constant of 0.15 N/m and a radius of curvature of <20 nm. AFM imaging was performed at room temperature (~25°C) on freshly cleaved mica surfaces. A liquid flow cell (glass probe holder, MTFML, Bruker Corporation) was used to scan the surfaces in a liquid environment and to exchange solution *in situ*. All images were recorded at a resolution of 512 × 512 pixels and with a scan rate of 1 Hz. The z-set point and differential gains were manually optimized during each scan. Images were analyzed and processed in the Gwyddion 2.22 software.

## Results

### Characterization of lipid composition and vesicle formation

Total lipid extracts were prepared from hydrogenated and deuterated *E*. *coli* bacteria (henceforth referred to as hEcoli and dEcoli, respectively). The dEcoli bacteria were grown at 30°C following a protocol for adaptation to deuterated minimal media; hEcoli was grown at 37°C. Fractionation of both extracts by thin layer chromatography showed similar phospholipid composition for hEcoli and dEcoli, consisting of approximately 75% PE, 13% PG and 12% CL (Fig 1B). MALDI-TOF MS analysis showed that the majority of the PE and PG species contain 16:0 and 18:1 acyl chains (see Fig 1A and S1 Fig) including cyclic structures such as cyc17:0 in agreement with previous results.[54]

**Fig 2:**
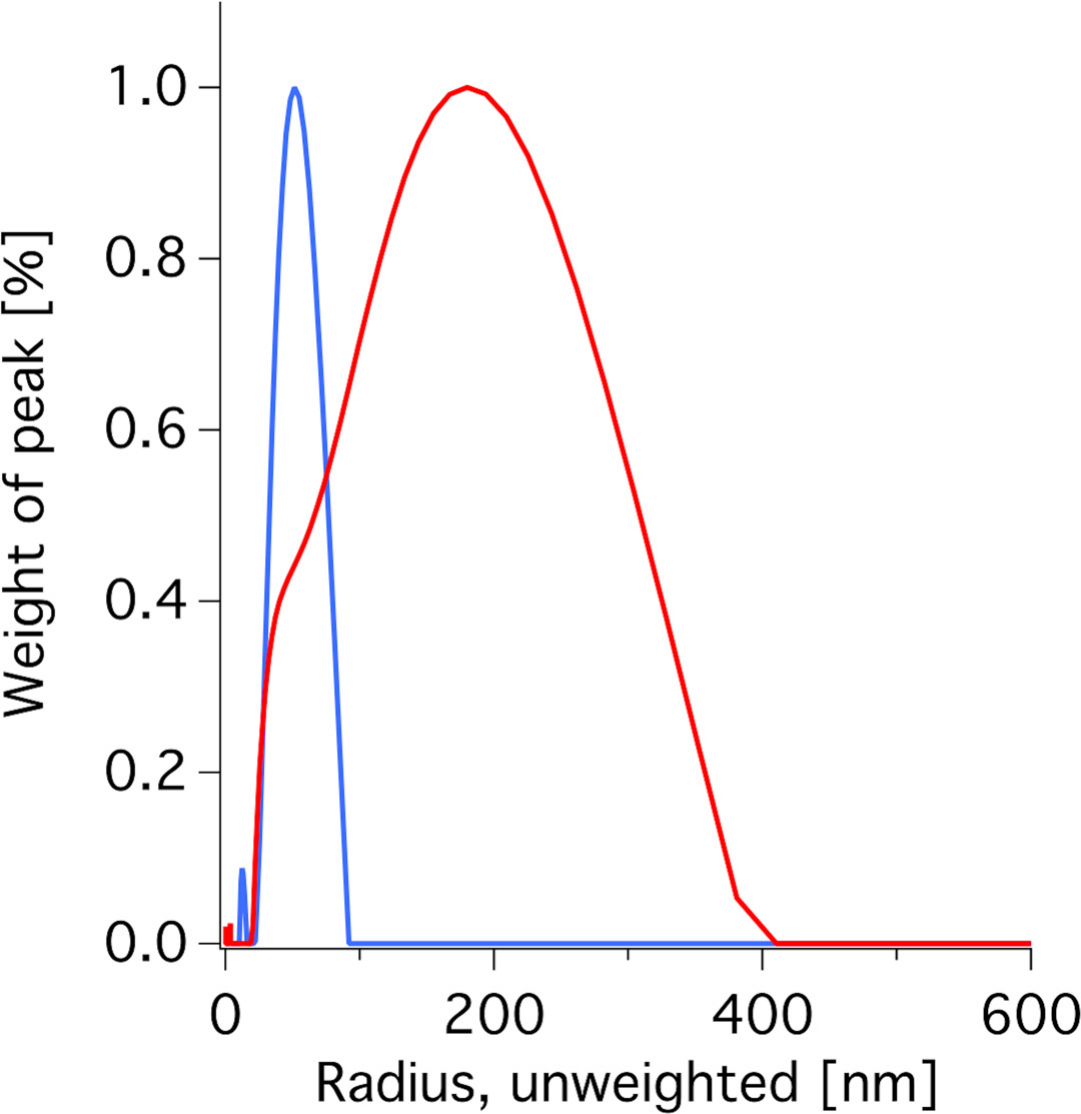
Size distribution of vesicles formed from hydrogenated *E*. *coli* lipids. Vesicles were formed by tip (blue) or bath (red) sonication and analyzed by DLS measurements at 90**°**.

The lipid extracts were used to form vesicles by bath sonication (1 h at 50°C) or tip sonication (20 min at 50°C). Dynamic light scattering showed that the size distribution depended considerably on the method of sonication (see Fig 2). Vesicle size has significant impact on the QCM-D responses for bilayer formation by vesicle fusion.[55, 56] In particular, small vesicles (<90 nm) seem to have a higher propensity of fusing than larger vesicles, even for simple POPC vesicles in the fluid phase.[55] If a significant population of large vesicles is present in the sample solution, these can stay attached as intact vesicles on top of the bilayer and cause large signals in both frequency and dissipation (see discussion below). The presence of bound vesicles is not desired since it complicates the interpretation of the data[39]. Therefore it is of high importance to ensure a population predominantly comprised by small unilamellar vesicles (SUVs). Thus, we used SUVs formed by tip-sonication until a clear solution was obtained for the subsequent formation of supported lipid bilayers.

### Assessment of supported bilayer formation by vesicle fusion using QCM-D

QCM-D is an *in situ*, label-free, acoustic technique, which allows for measuring real-time mass adsorption/desorption to/from a surface in a controllable (temperature, solution exchange) liquid environment. The QCM-D response includes the change in frequency of an oscillating crystal (related to mass changes) and the change in dissipation of energy in the adsorbed layer (related to viscosity and elasticity changes).[57] In this way, formation of a bilayer via adsorption and fusion of SUVs can be followed *in situ*.[40, 58] In general, a decrease in frequency corresponds to mass adsorption, while an increase indicates mass desorption. On the other hand, an increase in dissipation corresponds to formation of a softer, more viscous layer, whereas a decrease in dissipation reflects a more rigid layer, which is well coupled to the sensor surface. The QCM-D is extremely sensitive to vesicles coupled to the sensor crystal, whether they are directly attached to the surface, adsorbed in bilayer defects or attached on top of an already formed bilayer. Therefore, it can be difficult to establish if a supported lipid bilayer is present at all when vesicles are co-adsorbed.[39] Low deposition temperatures have previously shown to induce a decrease in the rate of vesicle fusion, even for single lipids in the fluid phase.[56] For our lipid bilayer depositions, we prepared SUVs by tip sonication and performed the deposition at 50°C in order to minimize the effects of potential co-adsorbed vesicles,[39] as discussed above.

**Fig 3:**
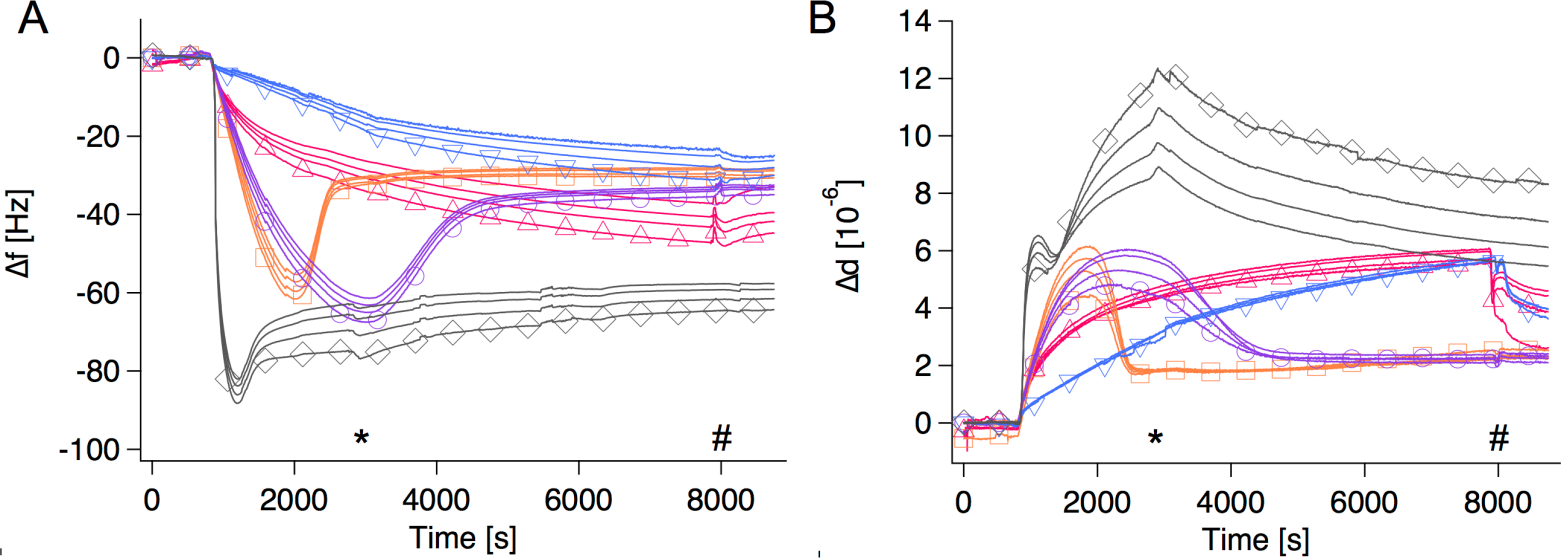
Selected QCM-D traces for bilayer formation, which were part of the optimization process. A: frequency changes. B: dissipation changes. Several overtones are shown for each experiment; 5^th^, 7^th^, 9^th^ and 11^th^. The 5^th^ overtone carries the marker. SUVs made from Avanti *E*. *coli* total lipid extract were added to clean silica under different experimental conditions: blue down-pointing triangle: 100 μg/ml lipid, 1 mM CaCl_2_, orange square: 100 μg/ml lipid, 2 mM CaCl_2_, purple circle: 50 μg/ml lipid, 2 mM CaCl_2_, gray diamond: 250 μg/ml lipid, 2 mM CaCl_2_, pink up-pointing triangle: 100 μg/ml lipid, 2 mM CaCl_2_,–pump stopped after initial adsorption. The * indicates the point where trace number 4 was rinsed with buffer. The others were rinsed at the point marked with a #.

In order to optimize the conditions for lipid bilayer formation from *E*. *coli* total lipid extracts we systematically varied lipid concentration, CaCl_2_ concentration, and flow rate. For the optimization process we used the commercially available *E*. *coli* total extract and the assessment was performed using QCM-D. From Fig 3 it is clear that the experimental conditions for bilayer deposition had profound effects on the QCM-D responses. Briefly, the concentration of CaCl_2_ in the buffer had great impact on the bilayer formation when using a lipid concentration of 100 mM under continuous flow: 1 mM CaCl_2_ led to impaired lipid deposition to the surface (blue down-pointing triangles), whereas 2 mM CaCl_2_ was enough to bridge between the anionic vesicles and the surface. Thus, this facilitates bilayer formation (orange squares). The lipid concentration was varied between 50–250 μg/mL to assess the optimal concentration where a bilayer could be formed within a reasonable time frame while at the same time avoiding a large amount of excess vesicles attaching to the bilayer.

**Fig 4:**
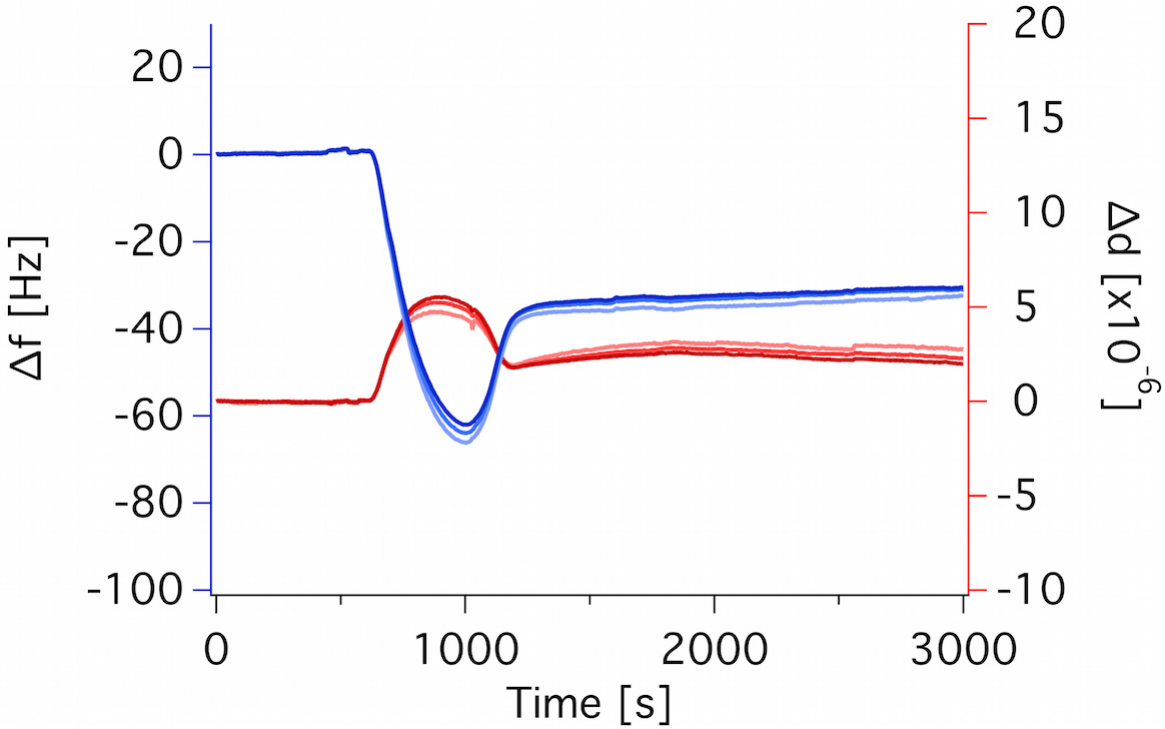
QCM-D traces for hEcoli bilayer formation under optimal conditions for SLB deposition. Deposition was done with 100 μg/mL lipid, 10 mM TRIS, 2 mM CaCl_2,_ 50°C, 100 μL/min. Overtones 5, 7 and 9 are shown increasing from light to dark hues. Frequency: blue, dissipation: red.

A lipid concentration of 50 μg/mL led to slow vesicle fusion (purple circles) while 250 μg/mL led to a significant amount of co-adsorbed vesicles (gray diamonds). However, these vesicles could be partly rinsed off with buffer (see kink in the responses, in particular for Δd, at the time marked with a star). The optimal lipid concentration was found to be 100 μg/mL, while the optimal CaCl_2_ concentration was found to be 2 mM (orange squares). Moreover, deposition under continuous flow is recommended for successful SLB formation even under these optimal conditions since QCM-D traces proceeded with very slow kinetics and with the lack of the expected changes in frequency and dissipation signal if the flow was stopped after the first adsorption response (pink up-pointing triangles). Finally, these optimal conditions led also to successful SLB formation via vesicle fusion of the *E*. *coli* lipids extracted in-house (see example of QCM-D traces for hEcoli in Fig 4).

### Structural analysis of *E*. *coli* supported lipid bilayers by NR and AFM

**Fig 5:**
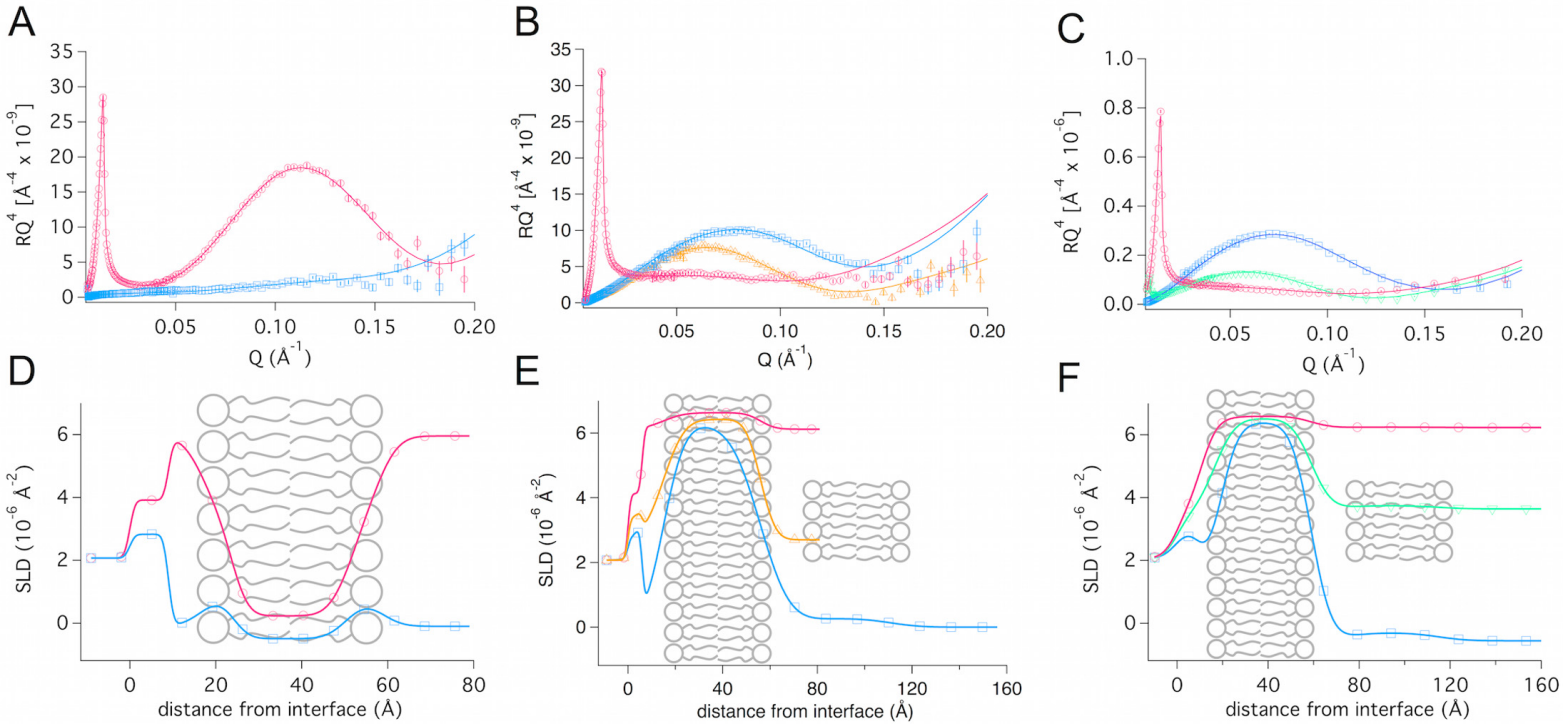
Neutron reflection intensity as a function of scattering vector, Q. (A) hEcoli deposited at 50°C, (B) dEcoli deposited at 50°C and (C) dEcoli deposited at 25°C. The contrasts used are: H_2_O (blue squares), 40% D_2_O (yellow triangle pointing up) and 60% D_2_O (green down-pointing triangle) and pure D_2_O (pink circles). All reflectivity profiles were measured at 25°C regardless of deposition temperature. Panel D-F give the SLD profiles and bilayer sketches corresponding to the reflection curve above it. In E and F the small additional patch of bilayer to the right represents the extra bilayer of very low coverage, which is used as a model for a small amount of co-adsorbed vesicles. In E, this extra layer was only present in the first contrast (H_2_O) measured and, thus, only the blue SLD profile represents the extended model.

Neutron reflection measurements were carried out on supported lipid bilayers formed by vesicle fusion of hEcoli and dEcoli extracts in order to obtain structural information on the bilayers. The bilayers were deposited at 50°C and measured at 25°C or deposited and measured at 25°C. The reflection profiles, including best fits for the hydrogenated and per-deuterated membrane, can be found in Fig 5A and 5B while the parameters for the best fits are shown in Table 1A and 1B. The hEcoli membrane (A) was fitted using a symmetric three-layer model as typically done for lipid bilayers.[39, 52, 59] The headgroup sizes were found to be 7 ± 1 Å, while the size of the tail region was 27 ± 1 Å.

**Table 1:** Neutron reflection fitting parameters for hEcoli deposited at 50°C (A) and dEcoli deposited at 50°C (B) or 25°C (C). In the case of dEcoli it was necessary to add a vesicle layer of low coverage (light grey shadings). These vesicles were rinsed away by contrast changes in bilayer B but not in bilayer C. The fitted parameters are: d = thickness, solvent%: volume fraction of the solvent, SLD = the fitted scattering length density of the layer, where (a) and (b) correspond to the headgroup SLD in H_2_O and D_2_O respectively. The asterisks (*) signify the layer closest to the silica surface.

**A: hEcoli deposited at 50°C and measured at 25°C**					
**Layer**	**d [Å]**	**Solvent [%]**	**SLD**	**Interfacial roughness [Å]**	**Notes**
Water*	8 ± 1	100	-	4 ± 1	
**Head**	7 ± 1	35 ± 3	1.55^a^/2.16^b^	4 ± 1	
**Tail**	27 ± 1	12 ± 1	-0.55	4 ± 1	
**Head**	7 ± 1	35 ± 3	1.55^a^/2.16^b^	4 ± 1	
**TOTAL**	41 ± 3				
**B: dEcoli deposited at 50°C and measured at 25 °C**					
Water*	9 ± 1	100	-	7 ± 2	
**Bilayer**	41 ± 1	6 ± 1	6.66	10 ± 1	
***Water***	*14 ± 1*	*100*	*-*	*10 ± 5*	
***Bilayer***	*41 ± 5*	*96 ± 1*	*6*.*66*	*10 ± 5*	*Vesicles present only for the first contrast (H*_*2*_ *O)*.
**C: dEcoli deposited and measured at 25°C**					
Water*	6 ± 1	100	-	7 ± 2	
**Bilayer**	42 ± 1	3 ± 1	6.60	7 ± 1	
***Water***	*14 ± 1*	*100*	*-*	*10 ± 5*	
***Bilayer***	*42 ± 5*	*97±1*	*6*.*60*	*10 ± 5*	*Vesicles present in all three contrasts*.

For the per-deuterated dEcoli membrane (Fig 5B), good fits were obtained using a one-layer model that contained an additional vesicle layer of very low coverage (4 ± 1% v/v). The SLB layer was 41 ± 1 Å thick and had a roughness of 7 ± 1 Å, the latter being on the same size range as the hEcoli headgroup. The vesicle layer was fitted as two extra layers (marked by italics in Table 1) that represented a water layer and a single lipid bilayer of low coverage. Due to the size polydispersity of the few bound vesicles and the softness of the material, the upper lipid layer becomes diffuse and thus the reflection profile is sensitive only to the flat part of the bound vesicles, which is aligned with the supported lipid bilayer.

This approach of modeling a vesicular layer was done previously for 1,2-dipalmitoyl-*sn*-glycero-3-phosphocholine (DPPC) bilayers deposited at 25°C.[39] Even though the vesicle layer comprised a mere 4 ± 1% v/v in coverage, it was a necessary addition to the model to achieve a good fit to the data. Successful bilayer formation was also obtained at 25°C leading to a membrane with similar structural properties than those formed at 50°C–see Fig 5C and fitting parameters in Table 1C. The attached vesicles were removed by rinsing due to the contrast change from H_2_O to 40% D_2_O for the dEcoli bilayer deposited 50°C (Table 1B) while they remained attached on the dEcoli bilayer deposited at 25°C (Table 1C).

**Fig 6:**
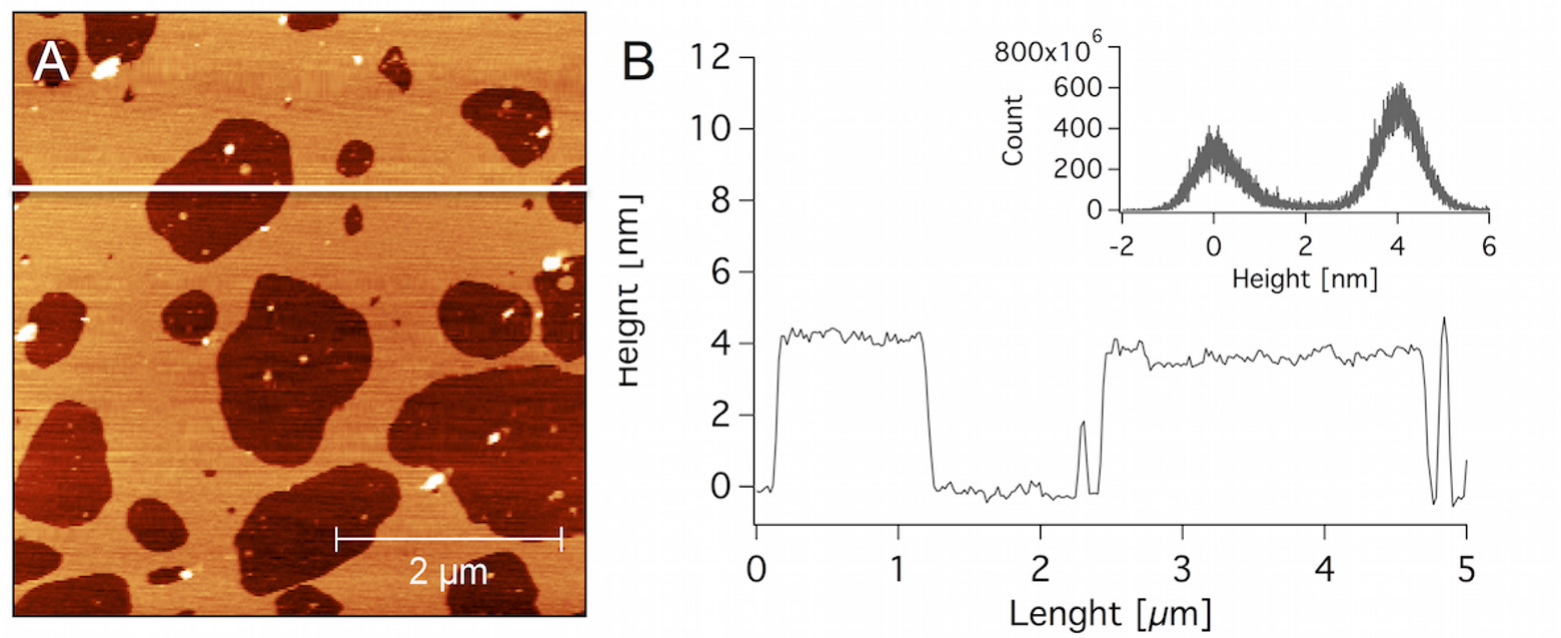
AFM image of a supported lipid membrane of *E*. *coli* lipids. A: hEcoli membrane of low coverage. B: a height profile corresponding to the white line in A. Inset in B shows the overall height distribution.

The hEcoli thickness fitted from the NR measurements was confirmed by AFM imaging. In general, high coverage lipid bilayers were formed with a small number of vesicles attached. Fig 6A presents an image of an incomplete membrane, from which the thickness could be measured. A height distribution centered at 4 nm and a linear height profile can be found in Fig 6B. This thickness is similar as to what has been reported for a ternary system composed of POPE/POPG/CL[32] and for *E*. *coli* polar extracts[60].

## Discussion

Our NR data shows that SLBs formed via vesicle fusion from total *E*. *coli* lipid extracts present high coverage and structural features similar to fluid phosphatidylcholine membranes made from lipids with 16:0 acyl chains (fluid DPPC; tail: 28 ± 1, head: 7 ± 1 Å [39, 61]). The typical chain lengths of the *E*. *coli* extracts are C16, C17_cyc_ and C18 whereby slight differences between PE and PG can be seen (see MS spectra in S1 Fig). The reconstituted membranes are slightly thinner than supported lipid membranes based on phosphatidylcholine membranes with 16:0–18:1 acyl chains (1-palmitoyl-2-oleoyl-*sn*-glycero-3-phosphocholine (POPC), tail: 31 ± 1 Å, head 8 ± 1 Å) as measured by neutron reflection.[52, 61] PE lipids are known to increase the chain order of lipid mixtures and thus slightly increase the hydrophobic thickness (by 1 Å) of lipid bilayers.[31] However, this effect could be counteracted by the presence of cyclic species that decrease the chain order. Molecular dynamics simulations carried out by Pandit *et al*., have shown that the cyclic lipid species found in bacterial membranes result in thinner and more rigid membranes than their aliphatic counterparts.[62]

Asymmetric model membranes formed by a single saturated LPS and DPPC were previously shown to have a headgroup thickness of ~15 Å or more for an LPS content higher than 19%. For hEcoli we found headgroup thickness comparable to PC while the interfacial roughness of dEcoli is on the same size order.[63] Therefore the structural parameters obtained in our work suggest that the SLBs of total *E*. *coli* extracts do not comprise a large amount of LPS containing larger oligosaccharide residues. This is expected since longer oligosaccharides are rather soluble in water and therefore can be lost during the vesicle preparation procedure.

The difficulties of forming supported lipid bilayers of bacterial membranes are apparent from the scarcity of publications on the subject and the differing accounts of the rate of success published in literature so far. A survey of the literature shows that already in 1995 Nollert *et al*. was able to successfully form a supported vesicle layer of *E*. *coli* lipids via vesicle fusion, and observed using fluorescence microscopy that the lipid vesicles fused upon addition of divalent cations to the vesicles.[42] Since that time, few studies of the formation of supported bilayers composed of lipids extracted from bacteria (isolated inner[64] and outer membranes,[65] commercially available polar extracts[13, 14, 60] and total lipid extracts[15]) were published and some discrepancies exist in literature as to whether or not vesicle fusion of such complex mixtures can be completed with success. This seems to be partly a consequence of the methods used to study lipid bilayer formation and the structure of the resulting membrane, as well as to the experimental conditions used and the method of preparing the lipid vesicles. Domenech *et al*. showed by AFM imaging that bilayers of *E*. *coli* polar lipid extracts successfully formed via vesicle fusion on mica.[14, 60] Nevertheless, others have concluded that it is not possible to form complete membranes on SiO_2_. In particular, Merz *et al*.[15] studied SLBs formed by fusion of vesicles composed of *E*. *coli* total lipid extract in detail including a thorough account for the effect of choice of substrate and divalent cation concentration. The complex total lipid extract containing the entire range of bacterial lipids was believed to adopt a non-planar geometry in contrast to the simpler two-component mimics based on 1-palmitoyl-2-oleoyl-*sn*-glycero-3-phosphoethanolamine (POPE) and 1-palmitoyl-2-oleoyl-*sn*-glycero-3-phosphoglycerol (POPG), which were concluded to be planar. They reported only partial bilayer formation on SiO_2_, while higher bilayer coverage was found for deposition onto TiO_2_. These findings were based on QCM-D, optical waveguide lightmode spectroscopy (OWLS) and fluorescence recovery after photobleaching (FRAP). Similarly, Dodd *et al*. extracted lipids from native *E*. *coli* inner membranes (including membrane associated proteins) and found, using QCM-D, that SLBs could be formed only by mixing of these lipids with significant amounts of POPC. [64] These authors concluded that 20–40% inner membrane lipids caused QCM-D traces that indicated some vesicle adsorption, while 60% or more led to QCM-D responses, which gave no indication of vesicle rupture. However, their AFM images confirmed the presence of SLBs with varying degrees of attached vesicles. They also found that SLB formation was improved by elevating the temperature to 35°C, however, in their study all depositions were done without a fusion promoter.

We have previously discussed the use of QCM-D for evaluating lipid bilayer deposition via vesicle fusion.[39] QCM-D is a very powerful technique, but the extremely high sensitivity (nanograms) of adsorbed material, coupled water and especially water-filled vesicles co-adsorbed with a membrane can lead to signals which completely mask the responses typically related to bilayer formation. Co-adsorption of vesicles in membrane defects or on top of the bilayer seems particularly prominent for complex mixtures and for lipid mixtures where a fusion promotor is needed. We have now demonstrated that the cation concentration, the lipid concentration and flow all together determine the success of forming SLBs of complex mixtures *in situ* via the vesicle fusion method. Once high-quality bilayers are formed in the QCM-D, the nanogram sensitivity of the instrument allows for probing of subtle changes in mass and viscosity changes as a result of biomolecule interactions.

## Conclusion

Using NR, AFM and QCM-D, we have established a protocol for formation of lipid bilayers composed of lipids extracted from *E*. *coli*. The vesicle preparation method, vesicle size, divalent cation concentration and temperature had a profound impact on the success of forming complete membranes as assessed by QCM-D. The method was further validated for various extract sources including total *E*. *coli* lipid extracts from hydrogenated and per-deuterated *E*. *coli* sources. Similar structures were obtained for hydrogenated and per-deuterated mixtures. These supported bacterial membranes serve as complex biosensors for biomolecule interactions and the per-deuterated extract, in particular, makes a powerful tool for neutron reflection studies e.g. of antimicrobial drug interaction due to the high contrast between the biomolecule and the deuterated lipid.

## Supporting Information

S1 Fig Negative ion mass spectrum of *E*. *coli* extract. Only the mass range of the phosphatidylethanolamine (PE) and the phosphatidylglycerol (PG) species is shown while the cardiolipin is omitted due to the complexity of the spectrum of this lipid class. All species are labeled by the m/z ratio and peak assignments as well as the relative amounts of the different fatty acyl residues are summarized in the table shown at the top of the figure. Identical data are obtained when the individual PE and PG fractions (isolated by TLC) were investigated.
(TIFF)
